# Can Chitin and Chitosan Replace the Lichen *Evernia prunastri* for Environmental Biomonitoring of Cu and Zn Air Contamination?

**DOI:** 10.3390/biology9090301

**Published:** 2020-09-19

**Authors:** Stefano Loppi, Andrea Vannini, Fabrizio Monaci, Daniel Dagodzo, Felix Blind, Michael Erler, Stefan Fränzle

**Affiliations:** 1 Department of Life Science, University of Siena, 53100 Siena, Italy; andrea.vannini@unisi.it (A.V.); fabrizio.monaci@unisi.it (F.M.); daniel.dagodzo@student.unisi.it (D.D.); 2 Technical University of Dresden, 01069 Dresden, Germany; felix.blind@gmx.de (F.B.); michaelerler@freenet.de (M.E.); stefan.fraenzle@tu-dresden.de (S.F.)

**Keywords:** bioaccumulation, biopolymers, biosorption, Cu, ion exchange, Zn

## Abstract

This study compared the ability of the lichen *Evernia prunastri*, chitin and chitosan to take up Cu^2+^ and Zn^2+^. It was hypothesized that chitin and chitosan have an accumulation capacity comparable to the lichen, so that these biopolymers could replace the use of *E. prunastri* for effective biomonitoring of Cu and Zn air pollution. Samples of the lichen *E. prunastri*, as well as chitin (from shrimps) and chitosan (from crabs), were incubated with Cu and Zn solutions at concentrations of 0 (control), 10, 25, 50, 75, and 100 µM and analyzed by Inductively Coupled Plasma Mass Spectrometry (ICP-MS). Metal concentrations accumulated by lichen, chitin and chitosan samples were strongly and linearly correlated with the concentrations in the treatment solutions. The lichen always showed significantly higher accumulation values compared to chitin and chitosan, which showed similar accumulation features. The outcomes of this study confirmed the great effectiveness of the lichen *Evernia prunastri* for environmental biomonitoring and showed that chitin and chitosan have a lower accumulation capacity, thus suggesting that although these biopolymers have the potential for replacing *E. prunastri* in polluted areas, their suitability may be limited in areas with intermediate or low pollution levels.

## 1. Introduction

The use of lichens for biomonitoring of trace metal air pollution is well established [1] and accepted to the point that these organisms are also used in environmental forensics [2,3] and decision-making processes [4]. Lichens do not have a root system and their mineral nutrition depends mainly on atmospheric inputs; they lack protective structure such as a cuticle and stomata, and trace metals can accumulate in their thalli up to levels well in excess of metabolic requirements [5]. Excluding particle interception, metal accumulation by lichens involves dynamic processes of uptake and release until an equilibrium with the surrounding environment is reached [6]. Accumulation of metal ions at extracellular binding sites on the cell wall by passive mechanisms is a well-documented, reversible process, largely dependent on the nature of the exchange sites and the affinity of the metal ions for these sites [7,8].

The major mechanisms of metal accumulation in lichens are particle deposition and extracellular ion exchange, which may account for up to 95% of the total [9]. Therefore, biomonitoring of airborne metal pollution by lichens mostly relies on these two mechanisms. This is also confirmed by: (i) the positive correlation between the elemental contents in the bulk deposition and in the thalli [10] and (ii) the similarity in the metal accumulation capacity of living and dead (devitalized) thalli in some transplant experiments [11]. Intracellular accumulation is commonly very limited, since excessive concentration of metals into the cell may cause severe damage [5]. The partitioning of extra- and intracellular metal in lichen thalli depends on the species and on the element [12,13]. For example, lichens demonstrate the ability to accumulate high concentrations of Cu and Zn extracellularly [14,15,16]. Both of these metals are of great environmental interest, being associated with atmospheric emissions from vehicular traffic [17]. For this reason, Cu and Zn have been commonly included in biomonitoring studies with lichens aimed at identifying and characterizing pollution sources in urban environments [18,19,20,21]. The opportunity and the convenience of environmental biomonitoring is seldom disputed with regard to some controversial issues, such as the definition of proper background values [22], the identification of the detection limit for a specific effect [23] and, above all, the large data variability [24], which is a possible cause of uncertainty in the results [25]. In addition, an ethical issue arise because, in large-scale and/or repeated surveys, a large amount of lichen material is required, which may drive a remarkable decrease in the lichen vegetation. This is also likely when using lichen transplants, where samples are collected from an unpolluted site and exposed elsewhere, with the risk of causing a dramatic reduction in the abundance of the selected species. Replacement of living organisms by passive samplers such as cellulose or quartz filters for the interception of airborne particles or cation exchange filters for the interception of elements in ionic form is simple and relatively inexpensive, although often not as effective as lichens [26].

Chitin (N-acetyl-D-glucosamine), after cellulose, is the second most abundant biopolymer worldwide, with an annual estimated biosynthesis of 100 million tons [27]. Together with its deacetylated form, chitosan, glucosamine-derived biopolymers are important components of the cell wall of fungi [28], as well as of several parts of crustaceans, insects, other arthropods, some bacteria, and mollusks [29]. Chitin and chitosan have a high adsorption capacity and, because of this, they have been widely used for the removal of toxic metals from wastewaters [30,31,32]. Fränzle [33] suggested using chitin for environmental biomonitoring and biogeochemical studies, and demonstrated the effectiveness of both the exoskeleton of living insects (cricket, *Gryllus assimilis*) and isolated chitin grafted on glass at taking up metals from different environmental media. Grafted chitin and chitin surfaces can be analyzed with minimal harm to organisms [33,34] because chitin can be dissolved in a mixture (solubility: 25–30 g chitin/L) of dimethylformamide (DMF), other carboxamides, and lactams, providing sizable amounts of lithium salts (e.g., chloride, nitrate, perchlorate, but not acetate). By adding water to the dissolving mixture, chitin re-precipitates as fibers or films without chemical changes. The Li^+^ solution in DMF is then put to the chitin surface and a thin layer is plainly removed, without producing cracks, holes, or craters. This extraction procedure should be repeated at least eight times [35].

While lichens have been tentatively used as biosorbents for wastewater remediation [36], the use of chitin and chitosan for monitoring airborne metals in ionic form remains unexplored. The aim of this study was to compare the ability of the lichen *Evernia prunastri*, chitin and chitosan to take up Cu^2+^ and Zn^2+^. The lichen species *Evernia prunastri* (L.) Ach., being widely used in field and laboratory studies [37,38,39,40], was chosen for the experiment because of its documented ability to bioaccumulate great amounts of trace elements and to reflect atmospheric bulk deposition [10,39,41]. In addition, the fruticose (shrubby) habitus of this epiphytic (tree-inhabiting) lichen allows easy handling of the thalli. We hypothesized that chitin and chitosan have an accumulation capacity comparable to the lichen, so that these biopolymers could replace the use of *E. prunastri* for effective biomonitoring of Cu and Zn air pollution.

## 2. Materials and Methods

### 2.1. Experimental Approach

Apparently healthy thalli of *E. prunastri* were harvested from a remote area of Tuscany, central Italy (43°11′60″ N, 11°21′33″ E, 310 m a.s.l.), far from any local source of air pollution. In the laboratory, extraneous material such as bark, insects, and other lichen species, were removed from the lichens with plastic tweezers and left to acclimate for 24 h in a climatic chamber at 15 ℃, RH = 65%, 40 µmol/m^2^/s photons PAR. Commercial chitin (from shrimps) and chitosan (from crabs) were used (Sigma-Aldrich, St. Louis, MO, USA).

Samples (ca. 200 mg) of the lichen and flakes of chitin and chitosan were separately put inside small nylon bags of ca. 8 cm^3^ (mesh 10 µm, side ca. 2 cm), that were first abundantly sprayed with deionized water to remove particles deposited onto the surface. The bags were then soaked by stirring for 1 h with individual Cu^2+^ and Zn^2+^ solutions provided as CuCl_2_ and ZnCl_2_ respectively, maintaining a constant 200/50 w/v ratio, at concentrations of 0 (control), 10, 25, 50, 75, 100 µM. These concentrations were selected by taking into account previous studies on element uptake in lichens [12] and are within the ranges of ecologically relevant levels found in polluted environments, such as urban and industrial areas [42]. The pH of the solutions was adjusted to 5.5 to maximize metal uptake [43]. After treatments, samples were rinsed three times for 5 s in deionized water to remove unbound ions simply deposited over the thallus surface. Samples were then allowed to air-dry on absorbing paper for 24 h in a climatic chamber, as described above, to allow possible later uptake [7]. The experiment was replicated independently three times.

### 2.2. Chemical Analysis

The total Cu and Zn content of whole lichen thalli, chitin and chitosan was measured by acid digestion in a microwave digestion system (Ethos 900, Milestone) using 3 mL of 70% HNO_3_, 0.2 mL of 60% HF and 0.5 mL of H_2_O_2_, and subsequent quantification by ICP-MS (Sciex Elan 6100, Perkin-Elmer, Waltham, MA, USA). One procedural blank and one sample of the certified material IAEA-336 ‘lichen’ were always included in the analysis. Recoveries were in the range 98–102% and precision of analysis, expressed as coefficient of variation of five replicates, was within 5% for all elements. Results are expressed on a dry weight basis.

### 2.3. Statistical Analysis

Linear regressions were run between metal concentrations in the treatment solutions and those accumulated by the lichen, chitin and chitosan to check for linear accumulation trends. Significance of differences (*p* < 0.05) between accumulated values in the three matrices were checked with the student *t* test, correcting for multiple comparisons according to Benjamini and Hochberg [44]. Data normality was checked with the Shapiro–Wilk test and equality of variances with the Levene test. All calculations were run using the free software R [45].

## 3. Results

**Figure 1:**
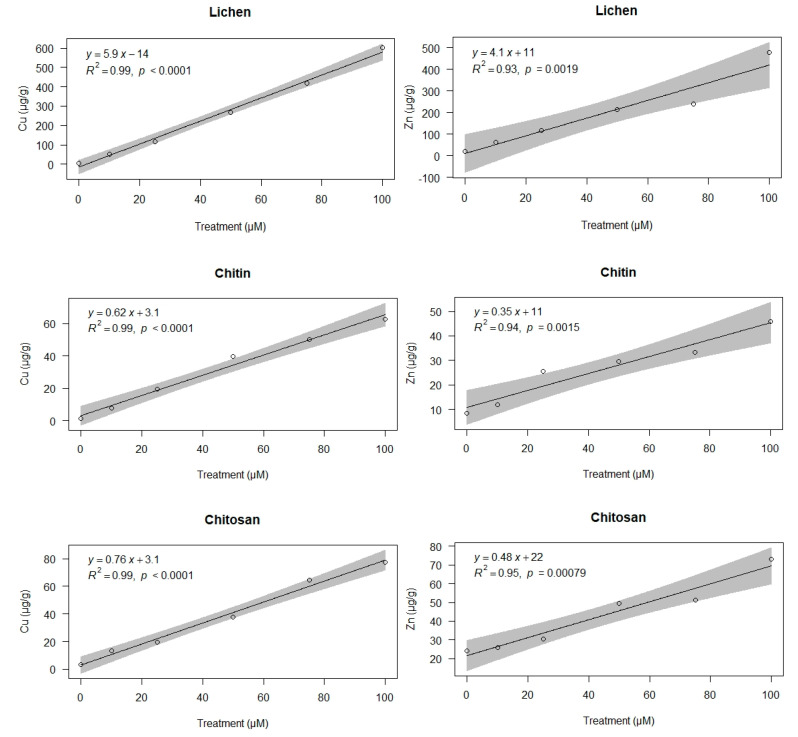
Copper and zinc concentrations (µg/g) in lichen, chitin, and chitosan samples after incubation with Cu (**left**) and Zn (**right**) solutions at the concentration 0 (control), 10, 25, 50, 75, and 100 µM. The grey area indicates the IC95 confidence interval.

Metal concentrations accumulated by lichen, chitin and chitosan samples after incubation with Cu and Zn solutions were strongly and linearly correlated with the concentrations in the treatment solutions (Figure 1). All regression models showed residuals with normal distributions and homogeneous variances. The slopes of the regression lines for lichen samples were always ca. 10-fold higher than those of chitin and chitosan, which were similar.

**Figure 2:**
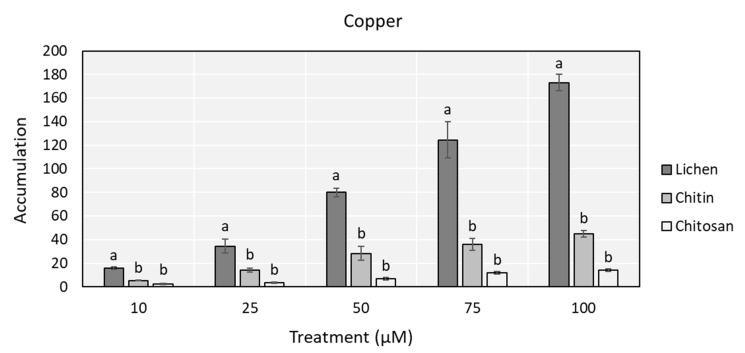

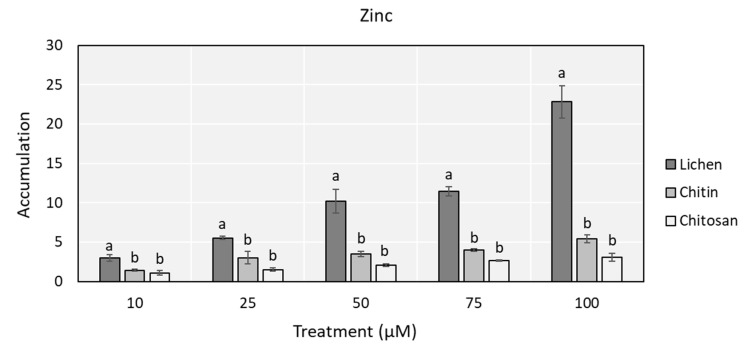
Copper and zinc accumulation (ratio between treated and control value ± SE) in lichen, chitin, and chitosan samples after incubation with Cu (up) and Zn (down) solutions at the concentration of 10, 25, 50, 75, and 100 µM. Different letters (a, b) indicate statistical significant differences between matrices (*p* < 0.05).

Control samples of lichen and chitosan showed similar concentrations for both Cu and Zn, while those of chitin were significantly lower (Table 1). For this reason, although differences in absolute concentrations were striking, to normalize for different starting concentrations, comparisons were made on data expressed as ratios to control values (Figure 2). Lichens always showed significantly higher accumulation values than chitin and chitosan, which presented similar accumulation features.

## 4. Discussion

Papers reporting on lichen biomonitoring surveys almost invariably state that the peculiarity of lichens to accumulate and tolerate huge amounts of toxic metals is especially related to their ability to trap airborne particles. As an example, Nieboer et al. [6] stated that “trapping of particulates contributes significantly to the elemental levels found in lichens”. Nevertheless, here we have clearly documented that accumulation in ionic form is also very important: when elements are provided in ionic form only, they are nicely and quickly accumulated proportionally to the amount available in the medium. On the contrary, there is plenty of literature reporting metal adsorption by chitin and chitosan from aqueous solutions (see e.g., [30,46]), but very little is known about their ability to intercept and trap metal-rich particles from dry deposition. Comparative data are available for some elements (Mn, Ni, La, Ce, Sm, Eu, Dy, and Yb) accumulated in chitin from the peel of *Pandalus borealis* shrimp and grafted chitin from the same source and particle attachment was observed in Ni solid phases of different kinds [47,48]. Dust particles can be retained by chitin, while also transferring ions: we studied this concerning nickel for both ground minerals (Millerite NiS, Nickelin NiAs) and insoluble Ni salts (oxalate, hexacyanoferrate(II), carbonate), and found sizable retention of Ni from these sources [47]. At normal pH, that is pH >> pzzp (point of zero zeta potential ≈ 3.5 for chitin), chitin is negatively charged; many particles found in the environment are positively charged and thus may stick to chitin. Whereas ion exchange normally does not produce fractionation, adsorption tendencies to chitin do vary with metal and oxidation state [49].

The binding capacity of chitin is about 40 µmol/g irrespective of the element [30,49,50]. The cation exchange capacity of *E. prunastri* is known to be ca. 150 µmol/g [51], roughly corresponding to ca. 40 µmol/g of lichen chitin, based on a weight share of chitin around 25–30%. Similar conclusions were reached by Puckett et al. [52], which showed that Cu saturation in lichens occurs at ca. 40 µmol/g. Thus, the binding capacity of lichens and chitin/chitosan is similar. Moreover, the differential metal affinity for Cu and Zn is also similar in lichens and chitin/chitosan, with Cu being much higher than Zn [6,53].

In lichens from unpolluted areas, it has been shown that the extracellular fraction of Cu and Zn, which are micronutrients, is as low as 5% and 1%, respectively [12]. The cellular fractionation may account for great differences in the starting condition for metal adsorption: in chitin and chitosan all Cu and Zn is adsorbed, while in lichens the amounts bound to the cell wall may be as low as 0.17 and 0.21 µg/g·dw, thus leaving a very high potential for metal binding. In addition, in living organisms, intracellular uptake may surely play an important role in sequestering important amounts of the provided elements, especially when they are soluble micronutrients [6]. In the lichen *Xanthoria parietina*, intracellular concentrations can represent up to 15–30% of total Cu and Zn, respectively [12]. Moreover, in addition to free chitin and chitosan, the cell wall of the lichen mycobiont is known to contain chitin– and chitosan–glucan complexes [54], which are the main structural polysaccharides of the cell walls of fungi [55,56]. Lastly, it is generally accepted that ion exchange is the dominant mode of uptake in lichens, while electrolyte sorption plays only a minor role [52].

All of the above reasons may account for a larger accumulation of Cu and Zn in lichens than in chitin and chitosan, as we have found in the present study. Metal uptake by chitin and chitosan is reported as determined by adsorption processes [30], with adsorption depending on several factors such as chitin/chitosan biomass and exposure time [31]. A biomass of 2–5 g/L of chitin was found to be optimal for maximum divalent metal ion removal [53], and our experimental conditions of 4 g/L are consistent with these values. The adsorption of metals to chitin is very fast, and it has been shown that full equilibrium is already reached after 30 min [53], and in some cases there is evidence that 10 minutes are enough for chitin-adsorption equilibrium except for a few elements, like Fe [35,48]. Our experimental time of 60 min was thus enough to warrant full adsorption equilibrium with metal ions in the treatment solutions.

## 5. Conclusions

This study confirmed that the lichen *Evernia prunastri* effectively accumulated Cu^2+^ and Zn^2+^ over a wide range of concentrations, further supporting the use of this lichen species for environmental biomonitoring. The accumulation capacity of chitin and chitosan was comparably lower, thereby suggesting that although these two biopolymers may, in fact, replace *E. prunastri*, this may be appropriate in areas with intermediate or low pollution levels.

## Figures and Tables

**Table 1:** Metal concentrations (mean ± SE, µg/g·dw) in control samples. Different letters (a, b) indicate statistically significant (*p* < 0.05) differences among matrices.

Element	Lichen	Chitin	Chitosan
**Cu**	3.4 ± 0.1 a	1.4 ± 0.4 b	3.2 ± 0.3 a
**Zn**	21 ± 2.5 a	8.4 ± 2.2 b	24 ± 2.1 a
